# Turquoise Urine in a Man Who Had Urinary Retention

**DOI:** 10.7759/cureus.47530

**Published:** 2023-10-23

**Authors:** Alan Lucerna, James Espinosa, Henry Schuitema

**Affiliations:** 1 Emergency Medicine, Jefferson Health - New Jersey, Stratford, USA

**Keywords:** reactions to medications, toxicology, urine discoloration, blue green urine, turquoise urine

## Abstract

While distressing to patients and physicians alike, urine discoloration is mostly benign. Most cases are due to food and drugs. A thorough history and physical exam generally elucidate the etiology but clinicians should have a broad knowledge of the differential diagnosis because life-threatening conditions, such as infection and poisonings, can also manifest as urine discoloration. Here, we present a case of a patient who presented with urinary retention and was found to have turquoise-colored urine, which was due to one of the patient's medications, Uribel. An appreciation of urine discoloration that is related to a benign and reversible medication can lead to stress reduction for patients and a reduction in unnecessary additional testing.

## Introduction

Most of what is known about the causes of urine discoloration comes from case reports. Formal research regarding this topic is lacking [1]. Patients are often surprised and alarmed when an abnormal urine color is observed, prompting thoughts of life-threatening diseases and severe internal organ dysfunctions. While distressing to patients and physicians alike, urine discoloration is mostly benign. Here, we present a case of a patient who presented with urinary retention and was found to have turquoise (blue-green) colored urine.

This case report was presented in poster form at Rowan University Research Day, on May 6, 2021.

## Case presentation

**Figure 1:**
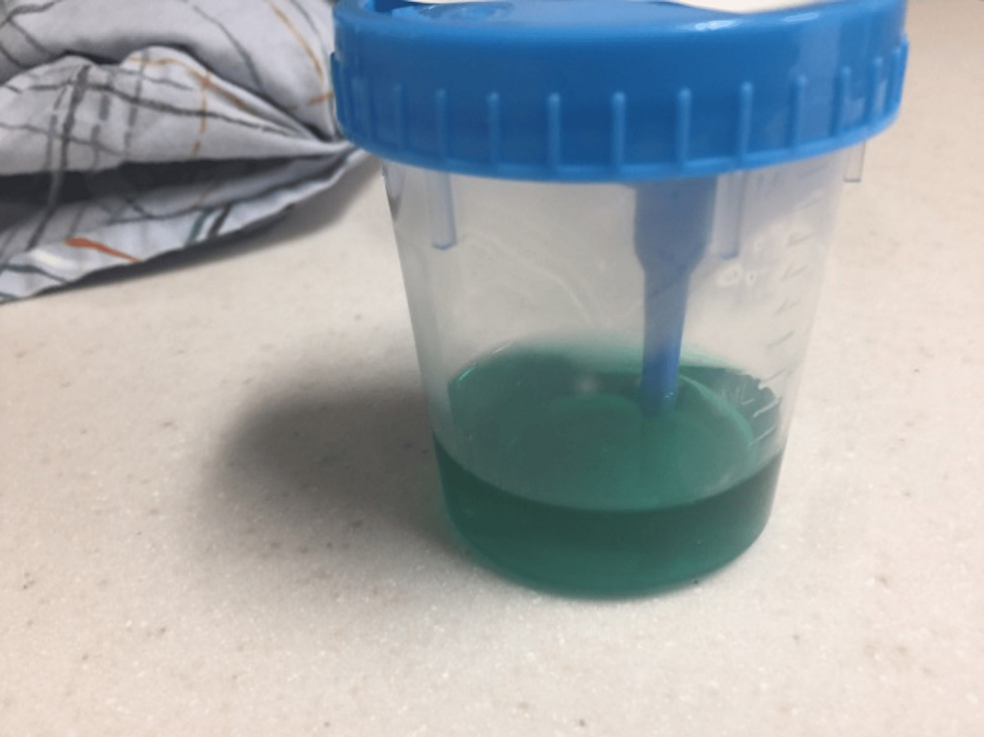
Urine sample showing turquoise color urine

**Figure 2:**
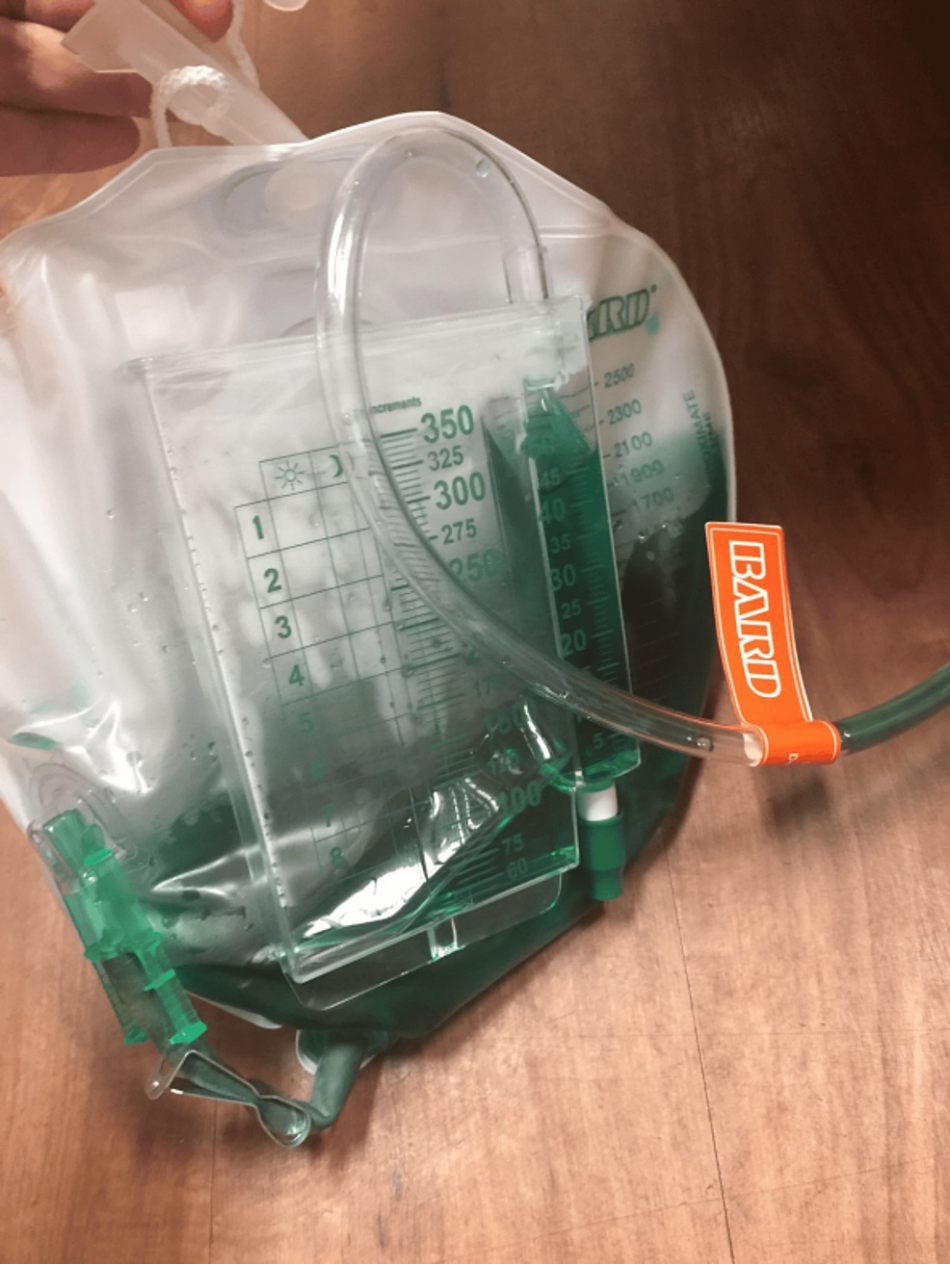
Foley bag showing turquoise color urine

A 75-year-old male presented to the emergency department (ED) for the evaluation of urinary retention and abdominal distention. He reported that he had not been able to urinate for several hours and had noticed abdominal distention. The patient had been evaluated in the ED five days prior for urinary hesitancy and distention and reported at both visits that he had a history of an enlarged prostate. At that time, he was diagnosed with bladder spasms. He denied fever or flank pain and appeared to be uncomfortable. A bladder scan showed greater than 800 cc of urine. His past medical history included an enlarged prostate, pancreatitis, valvular heart disease, and atrial fibrillation. His prior surgeries included an appendectomy, cholecystectomy, mitral valve surgery, rotator cuff surgery, and placement of an automatic implantable cardiac defibrillator. Vital signs on arrival were within normal limits. The patient had a distended abdomen with diffuse tenderness to palpation. A Foley catheter was placed without any difficulty. The Foley drainage bag filled easily with urine and the patient's symptoms resolved with urinary drainage. However, surprisingly, the urine color was turquoise (Figure 1). The patient's urine bag was also turquoise in color (Figure 2). The patient had not been previously aware of the change in urine color.

Basic laboratory studies, including a complete blood count, metabolic panel, and urinalysis, were obtained, with normal results.

What could have caused the patient’s urinary discoloration? A thorough review of his medications was done. The listed medications did not yield any possible source of urine discoloration. Finally, a call to the patient's urologist was made, and it was discovered that the patient had been prescribed Uribel, a medication the patient failed to report.

## Discussion

It is remarkable to consider that this case and the analysis of urinary color belong to one of the longest traditional medical approaches to the diagnosis of disease. Medical students hear of the work of Hippocrates in multiple contexts. He is an example of a pioneer in medicine who studied the color (and, as medical students have all heard, the taste) of urine as a diagnostic clue [2].

The usual yellow urine color is due to urochrome and to a lesser degree, urobilin and uroerythrin [2]. Aycock pointed out that deviations from the normal yellow urine color "can be distressing for patients, family members and physicians alike" and noted that there are many causes for urine discoloration, including blue-green (turquoise) urine. Most urinary color changes can be sorted out with a good history, as was seen in this case [1].

Medieval physicians developed a round disc (the matula) with various colors, against which a specimen could be compared [3-4]. Viswanathan reviewed the history of the urinary color observation and concluded that the urine bag is, in essence, a "modern-day matula" because urinary color - and the questions raised - is so often noted in the modern-day urine bag [2].

In the case described, the blue-green color was able to be attributed to the use of Uribel for benign prostatic hypertrophy. Uribel (methenamine, sodium phosphate monobasic, phenyl salicylate, methylene blue, and hyoscyamine sulfate) is a prescription medication used to decrease bladder spasms. It contains methylene blue, which is excreted as leucomethylene blue by the kidneys [2].

Other medications have been associated with blue-green urine, including amitriptyline and cimetidine [5]. Methylene blue installation into the bladder can result in several days of blue-green urinary discoloration. A urinary tract infection with Pseudomonas has been described as a cause of blue-green urine [5]. The jering plant is a protein-rich, low-carbohydrate food that is native to Southeast Asia. Jering is becoming more popular as a low-carbohydrate food. It can turn urine a blue or blue-green color [2]. Familial benign hypercalcemia is a rare, genetic metabolic disorder with an autosomal or X-linked recessive trait inheritance pattern, characterized by the incomplete intestinal breakdown of tryptophan. The syndrome can present with serious manifestations, including metabolic acidosis, recurrent hypoglycemia, and transient hepatopathy. One clue to the disease is that children with this disorder may have blue-green urine (or similarly stained diapers), and for this reason, familiar benign hypercalcemia is also known as Blue Diaper Syndrome [6].

An appreciation of urine discoloration that is related to a benign and reversible medication (such as green urine after propofol administration) can lead to stress reduction for patients and a reduction in unnecessary additional testing [7-8].

## Conclusions

Urinary discoloration is mostly due to benign etiologies. Gross examination of urine color provides clinicians with an opportunity to generate a broad differential diagnosis that, at times, can lead to the diagnosis of rare inherited disorders, systemic diseases, infections, and poisonings.

However, careful attention to the patient's medication list may provide the answer. An appreciation of urine discoloration that is related to a benign and reversible medication can lead to stress reduction for patients and a reduction in unnecessary additional testing.
